# Mapping evidence on the distribution of the costs associated with cancer of prostate, cervix, and female breast in the sub-Saharan Africa: protocol for a scoping review

**DOI:** 10.1186/s13643-021-01672-y

**Published:** 2021-04-17

**Authors:** Cebisile Ngcamphalala, Ellinor Ostensson, Mbuzeleni Hlongwa, Themba G. Ginindza

**Affiliations:** 1 Discipline of Public Health, School of Nursing and Public Health, University of KwaZulu-Natal, Mazisi Kunene Road, Durban, 4041 South Africa; 2 Department of Children’s and Women’s Health, Karolinska Institutet, Stockholm, Sweden; 3 Department of Medical Epidemiology and Biostatistics, Karolinska Institutet, Stockholm, Sweden

**Keywords:** Economic burden, Direct cost, Indirect cost, Cervical, Breast, Prostate, Treatment, Prevention, Societal, Neoplasm

## Abstract

**Background:**

Despite the well-documented information on cancer prevention and management, among noncommunicable diseases (NCDs), globally, cancer continues to be the second leading cause of morbidity and mortality with devastating economic consequences. The burden is disproportionately more among developing countries and the extent of evidence available on the economic consequences (direct and indirect costs) of cancer remains unknown in low-income countries particularly in the sub-Saharan region. Understanding the costs of illness is important to inform decision-making on setting up health care policies and informing economic evaluation of interventions.

This study aims to map evidence on the distribution of the economic burden (direct and indirect costs) associated with prevention, diagnosis, and treatment of three predominant cancers: prostate, cervix, and female breast in the sub-Saharan Africa.

**Methods:**

This scoping review will be reported according to the Preferred Reporting Items for Systematic Reviews and Meta-Analysis extension for Scoping Reviews (PRISMA-ScR), and will be conducted following Arksey and O’Malley’s framework. We will search PubMed/MEDLINE, Web of Science, CINHAL (via EBSCOhost platform), Science Direct, Cochrane Database of Systematic Reviews, Africa-Wide Information, Google Scholar, and WHO Library. We will perform hand-searching of the reference lists of included studies and other relevant documents.

Two reviewers will independently screen all citations, full-text articles, and abstract data.

We will include primary studies from all study designs reporting costs associated with prevention, diagnosis and treatment of prostate, cervical, and breast cancers in the sub-Saharan region. Data analysis will involve quantitative (e.g., frequencies) and qualitative (e.g., thematic analysis) methods. A narrative summary of findings will be presented.

**Discussion:**

This review will map the extent of information available on the economic burden (direct and indirect costs) of prostate, cervical, and breast cancers in the sub-Saharan region. Further guidance for future research in the subject area will be discussed.

**Systematic review registration:**

Open Science Framework

**Supplementary Information:**

The online version contains supplementary material available at 10.1186/s13643-021-01672-y.

## Background

Among the noncommunicable diseases (NCDs), cancer is ranked the second major causes of morbidity and mortality after cardiovascular diseases causing about 9.6 million deaths globally in 2018 [1]. This translates to about one cancer-related death in every six deaths [1]. Cancer disease is characterized by the transformation of normal cells into tumor cells in a multistage process that progresses from a pre-cancerous lesion to malignant tumor [1, 2]. The changes could be the result of interaction between individual genetic factors and three categories of external agents which are physical (ionization radiation), chemical (tobacco smoke and aflatoxin), and biological (infections) carcinogens [2, 3]. In addition, the global shift toward industrialized lifestyles is reported attributing to the rise in the cancers associated mainly with reproduction, dietary and hormonal risk factors [3].

While the world has made advances in prevention, diagnosis, and treatment of cancer, these have not yet translated into success in most countries particularly in developing countries [4]. Heterogeneity in the distribution of the disease burden magnitude and profile across and within regions (case fatality ratio) exist [4, 5]. Low- and middle-income countries (LMICs) bear higher proportions of cancer mortality than the proportions of incidence while the opposite is true for high-income countries (HICs) [1]. In part, the global variation in cancer incidence and mortality is attributable to lack of adequate preventive measures (screening and early diagnosis) and effective treatment facilities in LMICs compared to HICs [6]. Differences in the disease profile are also observed across regions. In the sub-Saharan region, most of the cancer-related deaths are attributable to cervical and prostate cancer, yet globally, lung cancer (among males) and breast cancer (among females) remain the leading cause of cancer-related mortality [4].

Cancer is associated with far more devastating economic effects than other conditions such as HIV and AIDS [7]. These include economic effects of premature deaths and disability from cancer as well as direct treatment cost (prevention, diagnosis, and treatment). In 2010, excluding direct cost, the burden was estimated at $1.16 trillion, representing more than 1.5% of global domestic product [7]. A study conducted in Eswatini, estimated annual cost associated with screening, managing, and treating cervical lesions at $12.6 million dollars in 2018 [8].

The sustainable development goals have set a target, specifically goal 1 and 3.4 which calls for end of poverty in all its forms and attainment of one-third reduction in noncommunicable diseases-related premature mortality by a third by 2030 [9]. This highlights the need for countries to understand the economic burden (direct and indirect costs) associated with cancers in order to inform resources allocation within countries. Direct costs consist of medical and non-medical costs incurred due to resource utilization because of inpatient and outpatient health care events associated with detection, treatment, and follow-up care of illness. These include transport costs and caregivers’ costs. On the other hand, indirect costs consist of productivity losses due to work absence (morbidity) and premature death from cancer (mortality) [10]. The sum of the two types of costs (direct and indirect) expresses the economic burden associated with illness.

There is limited knowledge on the extent of information available on the economic burden associated with the three predominant cancers (cervical, prostate, and breast) in LMICs particularly in the sub-Saharan Africa. A scoping review of the literature on the evidence on economic burden of the three cancers in the sub-Saharan African region will be conducted. The main aim is to map evidence on the costs (direct and indirect) associated with prevention, diagnosis, and treatment of three cancers in the sub-Saharan region.

## Methods

The present protocol has been registered within the Open Science Framework (https://osf.io/q5xdf/). The proposed scoping review will be reported in accordance with the reporting guidance provided in the Preferred Reporting Items for Systematic Reviews and Meta-analyses (PRISMA) extension for Scoping Reviews (PRISMA-ScR) (see checklist in Additional file 1) [11]. The scoping review methodology will be conducted in accordance with the framework proposed by Arksey and O’Malley [12]. The framework stage involves the following steps: (i) identifying the research questions, (ii) identifying relevant studies, (iii) study selection and eligibility, (iv) extracting and charting data, (v) collating, summarizing, and reporting results.

### Framework stage 1: Identifying the research question

The main research question is: “What is the distribution of evidence on the cost (direct and indirect) associated with cancer of the prostate, cervix and female breast in sub-Saharan Africa. These include costs associated with prevention, such as screening for all three cancers, diagnosis and management.”

*Research sub-questions include the following:*

- What is the direct medical cost incurred by the service provider as a result of interventions associated with cancer of the prostate, cervix, and female breast? These include costs associated with prevention including screening, diagnosis, treatment, transport, and caregivers for all three cancers?
- What is the cost for human papilloma virus (HPV) vaccine?
- What is the indirect cost associated with cancer of the cervix, breast, and prostate?
  - What is the average estimated years of life lost (YLL) due to cancer of the cervix, breast, and prostate cancer-related premature mortality?
  - What is the average estimated productivity losses due to work absence and premature mortality as a result of the cancer of cervix, breast, and prostate?

### Framework stage 2: Identifying relevant studies

Only literature published in English language will be considered. There will be no date restriction applied in the literature search. Cancer diagnosis remains a challenge in developing countries, applying time span in the searched publication might limit the number of studies available for consideration.

The primary source of literature will be a search of multiple electronic databases (from their inception onwards): PubMed/MEDLINE, Web of Science, CINHAL (via EBSCOhost platform), Science Direct, Cochrane Database of Systematic Reviews, and Africa-Wide Information. The secondary source of potentially relevant material will be a search of the gray or difficult to locate literature, including Google Scholar and WHO Library. We will perform hand searching of the reference lists of included studies, relevant reviews, reports, or other relevant documents. Content experts and authors who are prolific in the field will be contacted. The search will include a broad range of terms and keywords related to “costs,” “economic burden,” and “breast cancer,” “prostate cancer,” “cervical cancer,” and sub-Saharan countries. A draft search strategy for PubMed/MEDLINE is provided in Additional file 2. Where we identify systematic reviews, we will count the number of studies included in the review that potentially meet our inclusion criteria to note studies that could have been missed by our search.

### Framework stage 3: Study selection and eligibility

Identified studies with relevant title to the study topic will be uploaded to Endnote library version 7. Duplicates will be removed before analysis is conducted. Based on the eligibility criteria, an inclusion and exclusion criteria will be developed to eliminate studies that does not address the study question. The screening of relevant studies will be conducted by two independent reviewers using abstract screening form developed as per the eligibility criteria. Studies found irrelevant from the abstract will be eliminated and any discrepancies between the reviewers will be resolved through consensus involving a third reviewer. It is argued that abstract review cannot be considered as capturing the full scope of the article [13]; thus, full article review is ought to be conducted. In this review, full-article review will be conducted on articles meeting the inclusion criteria and discrepancies encountered by the reviewers will be resolved by involving a third screener.

#### Eligibility criteria

Studies will be selected according to the population, exposure, and outcomes (PEO) framework [14].

Population: We will include studies involving individuals (regardless of age) in the sub-Saharan African region (see Additional file 3 Countries in the sub-Saharan region). Exposures: The exposures of interest will be cancers of the prostate, cervix uteri, and breast (only for female).

Outcomes of interest: The primary outcome will be the costs associated with the prevention, diagnosis, and treatment of prostate, cervical, and breast cancers. We will include all type of costs, including direct and indirect costs. Direct costs include all the resources necessary for prevention, treatment, and cancer care. Indirect costs include resources lost due to inability to work.

Eligible studies will include all primary study designs (e.g., experimental studies, observational studies, and health economic modeling) published in English. No limitations will be imposed on publication status (unpublished studies will be eligible for inclusion) and study conduct period.

#### Reporting the screening results

Results of titles searched from different databases will be presented in a tabular format. A PRISMA flow chart showing details of studies included and excluded at each stage of the study selection process will be provided.

### Framework stage 4: Charting the data

To identify key issues, themes, and variables to answer the research question, data will be entered in a data charting form (see Additional file 4). The charting form will include the following sub sections: author(s) and date of publication, study title, aim(s) or research questions, study design, study setting, type of cancer, type of costs reported, other measures reported, key findings, and conclusions from the authors.

### Framework stage 5: Collating, summarizing and reporting the results

Studies will be grouped by types of cancer (prostate, cervix, and female breast) and type of costs (direct or indirect) they analyzed, setting, and study design along with key findings. Using descriptive statistics, findings will be summarized and analyzed within constructed themes and be presented in a narrative format.

## Discussion

The aim of the proposed scoping review is to map available evidence on the economic burden (direct and indirect cost) associated with prostate, cervix uteri, and female breast in the sub-Saharan region. Cancer continues to be among the top causes of death globally with the burden disproportionately more in developing countries particularly the sub-Saharan African region. The economic burden of disease consists of public health resources spent and productivity losses incurred by the patient/society due to the disease [10]. While there has been progress in reporting the economic burden of cancer globally, documentation of similar evidence in the sub-Saharan region is not clearly understood.

The results of the study will point the extent of information available on the economic burden of the three cancers in the sub-Saharan region. Understanding the economic burden of illness remains important for low-income countries particularly the sub-Saharan region faced with high disease burden against limited resources. This is in line with the Sustainable Development Goals set target, specifically 1 and 3.4 which calls for end of poverty in all its forms and attainment of one-third reduction in noncommunicable diseases (NCD)-related premature mortality by 2030 [9]. Limiting the search of studies to English language is a potential limitation for this study as we are likely to miss relevant studies conducted in other languages. Also, the lack of quality assessment of the included articles is recognized as another potential limitation of this study. However, scoping reviews usually do not appraise the quality of evidence in the primary research studies [11]. The findings, in a narrative format will address the implications for health care policy including discussion on national immunization programs in sub-Saharan countries on primary prevention of cervical cancer through HPV vaccines and future research. We will disseminate findings through conference presentations and publication in peer-reviewed journals. Any amendments made to this protocol when conducting the review will be outlined in the Open Science Framework and reported in the final manuscript.

## Supplementary Information

**Additional file 1.** PRISMA-P populated checklist.

**Additional file 2.** Draft search strategy for PubMed/MEDLINE.

**Additional file 3.** List of countries in the sub-Saharan Africa.

**Additional file 4.** Data charting form draft.

## Data Availability

All data to be analyzed and reported in this scoping review will be from published literature, which is already in the public domain.

## Abbreviations

AIDS: Acquired immune deficiency syndrome
HIV: Human immunodeficiency virus
HICs: High-income countries
LICs: Low-income countries
LMICs: Low- and middle-income countries
MMAT: Mixed method quality appraisal tool
NCDS: Noncommunicable diseases
PEO: Population, exposure and outcomes
PRISMA-P: Preferred reporting items for systematic reviews and meta-analysis protocols
SDGs: Sustainable development goals

## References

[1] Bray F, Ferlay J, Soerjomataram I, Siegel RL, Torre LA, Jemal A (2018). Global cancer statistics 2018: GLOBOCAN estimates of incidence and mortality worldwide for 36 cancers in 185 countries.

[2] WHO. Cancer key facts. 2017.

[3] WHO (2013). Global action plan for the prevention and control of noncommunicable diseases 2013-2020.

[4] Kingham TP, Alatise OI, Vanderpuye V, Casper C, Abantanga FA, Kamara TB, Olopade OI, Habeebu M, Abdulkareem FB, Denny L (2013). Treatment of cancer in sub-Saharan Africa. Lancet Oncol.

[5] Bray F, Jemal A, Grey N, Ferlay J, Forman D (2012). Global cancer transitions according to the Human Development Index (2008–2030): a population-based study. Lancet Oncol.

[6] Ginsburg O, Bray F, Coleman MP, Vanderpuye V, Eniu A, Kotha SR, Sarker M, Huong TT, Allemani C, Dvaladze A, Gralow J, Yeates K, Taylor C, Oomman N, Krishnan S, Sullivan R, Kombe D, Blas MM, Parham G, Kassami N, Conteh L (2017). The global burden of women’s cancers: a grand challenge in global health. Lancet.

[7] Rijo J, Ross H. The global economic cost of cancer: American Cancer Society, Inc; 2010. http://phrma-docs.phrma.org/sites/default/files/pdf/08-17-2010_economic_impact_study.pdf.

[8] Ginindza TG, Sartorius B, Dlamini X, Östensson E (2017). Cost analysis of human papillomavirus-related cervical diseases and genital warts in Swaziland. PloS One.

[9] UNDP. Sustainable line Development Goals (SDGs): The United Nations Development Programme (UNDP); 2015. https://www.undp.org/content/dam/undp/library/corporate/brochure/SDGs_Booklet_Web_En.pdf.

[10] Jo C (2014). Cost-of-illness studies: concepts, scopes, and methods. Clin Mol Hepatol.

[11] Tricco AC, Lillie E, Zarin W, O’Brien KK, Colquhoun H, Levac D, Moher D, Peters MD, Horsley T, Weeks L (2018). PRISMA extension for scoping reviews (PRISMA-ScR): checklist and explanation. Ann Intern Med.

[12] Arksey H, O’Malley L (2005). Scoping studies: towards a methodological framework. Int J Soc Res Methodol.

[13] Badger D, Nursten J, Williams P, Woodward M (2000). Should all literature reviews be systematic?. Eval Res Educ.

[14] Lekoane BK, Mashamba-Thompson TP, Ginindza TG (2017). Mapping evidence on the distribution of human papillomavirus-related cancers in sub-Saharan Africa: scoping review protocol. Syst Rev.

