# Supplementary material for: Mapping evidence on the distribution of the costs associated with cancer of prostate, cervix, and female breast in the sub-Saharan Africa: protocol for a scoping review
Source: Syst Rev. 2021 Apr 17;10:113. doi: 10.1186/s13643-021-01672-y (PMC8052831; doi:10.1186/s13643-021-01672-y)
Supplement: Supplementary file 2 — Additional file 2. Draft search strategy for PubMed/MEDLINE. [file 13643_2021_1672_MOESM2_ESM.docx]

Additional file 2: Draft Search Strategy for PubMed/MEDLINE

| **PubMed** |
| --- |
| ("cervical "[MeSH Terms] OR "cervical"[All Fields]) AND cancer [All Fields] AND cost [All Fields]) OR ((“breast”[MeSH Terms] OR “breast” [All Fields]) AND cancer [All Fields] AND cost [All Fields]) OR ((“prostate”[MeSH Terms] OR “prostate” [All Fields]) AND cancer [All Fields] AND cost [All Fields])) OR ((“cervical”[MeSH Terms] OR "cervical"[All Fields]) AND neoplasm [All Fields] AND economic-burden [All Fields])) OR (“breast”[MeSH Terms] OR “breast” [All Fields]) AND neoplasm [All Fields] AND economic-burden [All Fields])) OR (“prostate”[MeSH Terms] OR “prostate” [All Fields]) AND neoplasm [All Fields] AND economic-burden [All Fields])) AND ("africa"[MeSH Terms] OR "africa"[All Fields])) OR "sub-Saharan Africa"[MeSH Terms] OR “sub-Saharan Africa”[All Fields])) OR "angola"[All Fields])) OR ("benin"[MeSH Terms] OR "benin"[All Fields])) OR ("botswana"[MeSH Terms] OR "botswana"[All Fields])) OR ("burkina faso"[MeSH Terms] OR ("burkina"[All Fields] AND "faso"[All Fields]) OR "burkina faso"[All Fields])) OR ("burundi"[MeSH Terms] OR "burundi"[All Fields])) OR ("cameroon"[MeSH Terms] OR "cameroon"[All Fields])) OR ("cabo verde"[MeSH Terms] OR ("cabo"[All Fields] AND "verde"[All Fields]) OR "cabo verde"[All Fields] OR ("cape"[All Fields] AND "verde"[All Fields]) OR "cape verde"[All Fields])) OR "Central African Republic"[All Fields]) OR ("chad"[MeSH Terms] OR "chad"[All Fields])) OR ("comoros"[MeSH Terms] OR "comoros"[All Fields])) OR "Congo brazzaville"[All Fields]) OR "Democratic republic of congo"[All Fields]) OR "Cote d'Ivoire"[All Fields]) OR ("djibouti"[MeSH Terms] OR "djibouti"[All Fields])) OR "Equatorial Guinea"[All Fields]) OR ("eritrea"[MeSH Terms] OR "eritrea"[All Fields])) OR ("ethiopia"[MeSH Terms] OR "ethiopia"[All Fields])) OR ("gabon"[MeSH Terms] OR "gabon"[All Fields])) OR ("gambia"[MeSH Terms] OR "gambia"[All Fields])) OR ("ghana"[MeSH Terms] OR "ghana"[All Fields])) OR ("guinea"[MeSH Terms] OR "guinea"[All Fields])) OR "Guinea-Bissau"[All Fields]) OR ("kenya"[MeSH Terms] OR "kenya"[All Fields])) OR ("lesotho"[MeSH Terms] OR "lesotho"[All Fields])) OR ("liberia"[MeSH Terms] OR "liberia"[All Fields])) OR ("madagascar"[MeSH Terms] OR "madagascar"[All Fields])) OR ("malawi"[MeSH Terms] OR "malawi"[All Fields])) OR ("mali"[MeSH Terms] OR "mali"[All Fields])) OR ("mauritania"[MeSH Terms] OR "mauritania"[All Fields])) OR ("mauritius"[MeSH Terms] OR "mauritius"[All Fields])) OR ("mozambique"[MeSH Terms] OR "mozambique"[All Fields])) OR ("namibia"[MeSH Terms] OR "namibia"[All Fields])) OR ("niger"[MeSH Terms] OR "niger"[All Fields])) OR ("nigeria"[MeSH Terms] OR "nigeria"[All Fields])) OR ("reunion"[MeSH Terms] OR "reunion"[All Fields])) OR ("rwanda"[MeSH Terms] OR "rwanda"[All Fields])) OR "Sao Tome and Principe"[All Fields]) OR ("senegal"[MeSH Terms] OR "senegal"[All Fields])) OR ("seychelles"[MeSH Terms] OR "seychelles"[All Fields])) OR ("sierra leone"[MeSH Terms] OR ("sierra"[All Fields] AND "leone"[All Fields]) OR "sierra leone"[All Fields])) OR ("somalia"[MeSH Terms] OR "somalia"[All Fields])) OR "South Africa"[All Fields]) OR ("sudan"[MeSH Terms] OR "sudan"[All Fields])) OR ("swaziland"[MeSH Terms] OR "swaziland"[All Fields])) OR ("Eswatini"[MeSH Terms] OR "Eswatini"[All Fields])) ("tanzania"[MeSH Terms] OR "tanzania"[All Fields])) OR ("togo"[MeSH Terms] OR "togo"[All Fields])) OR ("uganda"[MeSH Terms] OR "uganda"[All Fields])) OR "Western Sahara"[All Fields]) OR ("zambia"[MeSH Terms] OR "zambia"[All Fields])) OR ("zimbabwe"[MeSH Terms] OR "zimbabwe"[All Fields])) OR "west africa"[All Fields]) OR "east africa"[All Fields]) OR "southern africa"[All Fields] |
