# Supplementary material for: Mapping evidence on the distribution of the costs associated with cancer of prostate, cervix, and female breast in the sub-Saharan Africa: protocol for a scoping review
Source: Syst Rev. 2021 Apr 17;10:113. doi: 10.1186/s13643-021-01672-y (PMC8052831; doi:10.1186/s13643-021-01672-y)
Supplement: Supplementary file 3 — Additional file 3. List of countries in the sub-Saharan Africa. [file 13643_2021_1672_MOESM3_ESM.docx]

**Additional file 3: List of countries in the sub-Saharan region**

| **List of countries in the sub-Saharan region** | | |
| --- | --- | --- |
| Angola  Benin  Botswana  Burkina Faso  Burundi  Cabo Verde  Cameroon  Central African Republic  Chad  Comoros  Congo, Democratic Republic of  Congo, Republic of  Cote d'Ivoire  Equatorial Guinea  Eritrea  Eswatini (Formerly Known as Swaziland)  Ethiopia | Gabon  Gambia, The  Ghana  Guinea  Guinea-Bissau  Kenya  Lesotho  Liberia  Madagascar  Malawi  Mali  Mauritania  Mauritius  Mozambique  Namibia | Niger  Nigeria  Rwanda  Sao Tome and Principe  Senegal  Seychelles  Sierra Leone  Somalia  South Africa  South Sudan  Sudan  Tanzania  Togo  Uganda  Zambia  Zimbabwe |
