# Supplementary material for: Mapping evidence on the distribution of the costs associated with cancer of prostate, cervix, and female breast in the sub-Saharan Africa: protocol for a scoping review
Source: Syst Rev. 2021 Apr 17;10:113. doi: 10.1186/s13643-021-01672-y (PMC8052831; doi:10.1186/s13643-021-01672-y)
Supplement: Supplementary file 4 — Additional file 4. Data charting form draft. [file 13643_2021_1672_MOESM4_ESM.docx]

**Additional file 4. Data charting form draft**

| **Author(s)** | **Date of publication** | **Study title** | **Study design** | **Setting** | **Study aim (s)** | **Type of cancer assessed** | **Type of costs reported** | **Other measures reported** | **Key findings** | **Conclusion** | **Comments** |
| --- | --- | --- | --- | --- | --- | --- | --- | --- | --- | --- | --- |
| Goldie, S. J. Kuhn, L. Denny, L. Pollack, A. Wright, T. C. | 2001 | Policy analysis of cervical cancer screening strategies in low-resource settings: clinical benefits and cost-effectiveness Policy analysis of cervical cancer screening strategies in low-resource settings: clinical benefits and cost-effectiveness | Cost effectiveness-modelling study | South Africa | To assess alternative screening strategies for cervical cancer in developing countries and used country specific data to conduct policy analysis comparing clinical benefits and cost effectiveness of different cervical cancer screening strategies in black South African women | Cervical cancer | Lifetime costs | disability adjusted life year (DALY). | HPV testing followed by treatment of screening positive women at a second visit costed $39/YLS) cancer reduction, direct visual inspection (DVI) with immediate treatment of screen positive women at first visit was effective at (26% incidence reduction),Cytology followed by treatment of screen positive women at second visit was least effective . HPV DNA testing was generally more effective but more costly than DVI whilst DVI was effective and less costly than cytology | Cervical cancer screening strategies that in cooperate DVI or HPV DNA testing and eliminate colposcopy may offer attractive alternatives to cytology-based screening in low resource settings | Mathematical modelling study with hypothetical cohort assessing cost effectiveness of various cervical cancer interventions with multiple outcome measures. Ranging from lifetime costs, DALYS . Whilst the lifetime cost includes direct medical costs, but it did not provide comprehensive assessment of the costs, that is assessing direct and indirect costs of cervical cancer in SA. |
| Ginindza, T. G.  Sartorius, B.  Dlamini, X.  Östensson, E. | 2017 | Cost analysis of Human Papillomavirus-related cervical diseases and genital warts in Swaziland | Cost pf illness studies-cost analysis | Swaziland/Eswatini | To estimate the cost of HPV-related conditions such as cervical lesions, cervical cancer and genital warts. | Cervical cancer | Direct medical costs | N/A | The total annual estimated direct medical cost associated with screening, managing and treating cervical lesions, CC and genital warts in Swaziland was $16 million. The largest cost in the analysis was estimated for treatment of high-grade cervical lesions and cervical cancer representing 80% of the total cost ($12.6 million). Costs for screening only represented5% of the total cost ($0.9 million). Treatment of genital warts represented 6% of the total cost ($1million). | the economic burden of HPV-related cervical diseases and genital warts represents a major public health issue in Swaziland. Prevention of HPV infection with a national HPV immunization programmes for pre-adolescent girls would prevent the majority of CC related deaths and associated costs. | Study looked at the direct medical cost of cervical cancer in an elaborative manner compared to most costing studies reviewed in the Southern African region. Method was clearly defined, and focus was not on a particular intervention as it has been the case with most studies. Notably, the study was not conducted as part of a bigger study which made it to provide quite elaborate results . Annual costs estimations were presented from the providers. However, the study did not consider indirect costs |
|  |  |  |  |  |  |  |  |  |  |  |  |
|  |  |  |  |  |  |  |  |  |  |  |  |
